# Parent-Adolescent Communication, Self-Efficacy, and Self-Management of Type 1 Diabetes in Adolescents

**DOI:** 10.1177/26350106241304424

**Published:** 2025-01-10

**Authors:** Ella Tuohy, Pamela Gallagher, Caroline Rawdon, Nuala Murphy, Ciara McDonnell, Veronica Swallow, Veronica Lambert

**Affiliations:** School of Psychology, Dublin City University, Dublin, Ireland; School of Psychology, Dublin City University, Dublin, Ireland; School of Nursing, Psychotherapy and Community Health, Dublin City University, Dublin, Ireland; Diabetes and Endocrine Unit, Children’s Health Ireland at Temple Street, Dublin, Ireland; Diabetes and Endocrine Unit, Children’s Health Ireland at Temple Street, Dublin, Ireland; Trinity Research in Childhood Centre, School of Medicine, University of Dublin, Trinity College Dublin, Dublin, Ireland; College of Health, Wellbeing and Life Sciences, Sheffield Hallam University, Sheffield, England; School of Nursing, Psychotherapy and Community Health, Dublin City University, Dublin, Ireland

## Abstract

**Purpose:**

The purpose of this study was to investigate adolescent perspectives of parent-adolescent communication, type 1 diabetes mellitus (T1DM)-specific family conflict, self-efficacy, and their relationship to adolescent self-management of T1DM.

**Methods:**

A cross-sectional survey design was employed. Adolescents completed measures of parent-adolescent communication, T1DM-specific family conflict, self-efficacy, and self-management, which included activation and division of responsibility for management tasks.

**Results:**

Surveys were completed by 113 adolescents ages 11 to 17 years (mean age 13.85 years, SD 1.78) and living with T1DM for 6 months and longer. Hierarchical multiple regression sought to determine what variables make the most unique contribution to self-management of T1DM, division of family responsibility for management tasks, and activation. Self-efficacy was a significant predictor of division of family responsibility for T1DM management, patient activation, and all self-management subscales except collaboration with parents. Openness in parent-adolescent communication was a significant predictor of the diabetes communication and goals subscale of the self-management measure and activation. Problems in communication was a significant predictor of collaboration with parents and self-management goals.

**Conclusions:**

These findings suggest that family context characteristics, particularly parent-adolescent communication, and self-efficacy are important for engagement with self-management for adolescents living with T1DM. Findings can inform future family-focused self-management interventions to improve T1DM outcomes for adolescents living with T1DM.

For adolescents living with type 1 diabetes mellitus (T1DM), self-management includes collaboration with parents, performance of T1DM care activities, problem-solving related to T1DM, communication about T1DM, and possession of T1DM-related goals.^1^ During adolescence, adolescents are expected to become more responsible for T1DM self-management and assume an increasing amount of this responsibility from parents.^2^

Self-management is influenced by various factors, including family factors, which can serve as risk or protective factors in relation to their influence on proximal outcomes (eg, engagement with self-management tasks) and distal health outcomes (eg, A1C and health status).^3^ Previous research has highlighted the association between parent-adolescent interactions, adolescent independence in T1DM management, and T1DM outcomes.^4-6^ Adolescents’ perceptions of how autonomy-supportive their parents are is also related to management of T1DM and blood glucose levels.^4,5^ Furthermore, family factors, such as division of family responsibility and family conflict specific to T1DM, have been linked to meeting blood glucose targets in adolescents living with T1DM.^6-8^

These links that have been identified between family context, metabolic outcomes, and T1DM management suggest that negative communication about T1DM between parents and adolescents may also be important in terms of T1DM outcomes during adolescence. Indeed, openness and problems in communication are important for promoting bonding between family members, adaptability in times of stress, and overall, more optimal family functioning.^9,10^ However, to our knowledge, to date, there is no research examining the relationship between openness and the extent of problems in parent-adolescent communication to adolescent self-management of T1DM. Furthermore, previous research, described previously on adolescent T1DM management, predominantly focuses on distal outcomes, such as quality of life and A1C, rather than proximal outcomes, such as engagement with self-management behaviors. A focus on both proximal and distal outcomes may provide a more comprehensive perspective on self-management that considers family factors as they relate to engagement with self-management behaviors during adolescence.

Self-management processes (eg, self-efficacy, self-regulation, and beliefs) are important factors in determining both proximal and distal health outcomes.^3^ Self-efficacy (the beliefs held by the adolescent in relation to self-management of their illness) is an important component in assuming self-management responsibilities for adolescents living with chronic illness.^11^ Supportive or nonsupportive parental feedback can promote or undermine self-efficacy in self-management of T1DM during adolescence.^12^ Moreover, adolescents’ perspectives on the quality of their relationship with their parents has been found to be associated with some aspects of self-management.^13^ This suggests that parent-adolescent interactions and communication may influence both self-efficacy and self-management for adolescents living with T1DM. However, previous research has highlighted the need to examine how contextual factors, such as the family environment, and process variables, such as self-efficacy, are implicated in self-management outcomes for adolescents living with T1DM.^14^

Activation is a specific self-management behavior that encompasses knowledge, skills, and confidence for carrying out self-management.^15^ Activation has been associated with increased engagement with positive health behaviors, with highly activated individuals taking a more active role in managing their health.^16^ However, activation remains an understudied construct among adolescents.^17^ Given that adolescents are required to assume a more prominent role in T1DM management over time, there is a need to quantitatively examine whether family interactions contribute to adolescent activation in the context of adolescent involvement in self-management of T1DM.

In summary, there is a dearth of research examining the interplay between individual and family contextual factors and engagement with self-management in adolescents. Because research indicates that adolescents struggle with the negotiation of the demands of balancing self-management responsibilities with parental involvement, research seeking to understand how the process of becoming independent in T1D self-management occurs for adolescents is required. Furthermore, there is a need to examine proximal outcomes in addition to distal outcomes of self-management. Although research has advanced understanding into the importance of family factors for adolescent self-management of T1DM, little is known about the role of specific family context characteristics (ie, parent-adolescent communication and T1DM-specific family conflict) and self-management processes (self-efficacy) and their interrelationships to engagement with self-management. To address this gap, the purpose of this study was to investigate interrelationships between specific aspects of family context (parent-adolescent communication characteristics, T1DM-specific family conflict), self-management processes (self-efficacy), and self-management of T1DM. The specific hypothesis was more open parent-adolescent communication, fewer problems in parent-adolescent communication, less T1DM-specific family conflict, and higher T1DM self-efficacy will be associated with more adolescent T1DM self-management, higher levels of activation, and higher adolescent T1DM-related responsibility for T1DM management.

## Methods

### Design

This study is a cross-sectional survey. Ethical approval was granted by the Research Ethics Committees at the university (DCUREC/2018/012) and participating hospital study site (CHI/17.078).

### Sample

Participants were eligible if they were adolescents ages between 11 and 17 years and if they had a diagnosis of T1DM for 6 months or longer. Adolescents were ineligible if they also had any other significant medical condition and/or if they presented with a cognitive impairment or developmental delay.

### Recruitment

Adolescents were recruited through different routes. For example, adolescents who met the eligibility criteria were identified from outpatient clinic appointment lists by a nominated gatekeeper in the Diabetes and Endocrine Unit at a national pediatric hospital. Survey packs (ie, cover letter, parent and adolescent information sheets, adolescent questionnaire, and adolescent resource sheet) were posted to eligible adolescents living with T1D and their parents. Posters were also displayed and flyers distributed in the outpatient clinic to advertise the study, and interested participants could contact the researchers directly if they required more information on the survey. Parents of adolescents who had taken part in the previous qualitative study that is reported elsewhere^18^ and had agreed to be contacted to consider taking part in a future quantitative survey were also contacted by the research team. The qualitative study explored adolescent perspectives on communicating about self-management of T1DM and negotiating responsibilities for self-management with their parents.^19^ Semistructured interviews were conducted with 28 adolescents ages between 11 and 17 years. This qualitative study informed the selection of instruments and design of this quantitative cross-sectional survey. Open advertisements were placed on a national diabetes advocacy organization’s website and social media sites, where participants could access the study information, online survey link, and researchers’ contact details. For all recruitment routes, adolescents had the option of completing the questionnaire in hard-copy format or online. It was explained to all participants that return of a questionnaire signified parental consent and adolescent assent for the adolescent’s participation in the survey.

### Measures

Along with questions on demographic and clinical characteristics, 6 valid and reliable age-appropriate measures were included in the survey questionnaire.

The Parent-Adolescent Communication Scale (PACS) is a 20-item measure that measures openness and extent of problems in parent-adolescent communication on 2 subscales.^10^ Each subscale contains 10 items with responses on a 5-point Likert-type scale ranging from “strongly disagree” to “strongly agree.” The alpha coefficient for openness of communication is 0.87 and for problems in communication is 0.78.^10^ Higher scores on openness indicate more openness in parent-adolescent communication, and higher scores on problems indicate low problems in parent-adolescent communication. Higher scores for total communication indicate better parent-adolescent communication.

Family conflict related to T1DM was measured using the Diabetes Family Conflict Scale-Revised–Youth Version (DFCS-R).^20^ The DFCS-R is a 19-item scale measuring T1DM-specific conflict around direct management tasks (eg, checking bloods, administering insulin) and T1DM-specific conflict around indirect management tasks (eg, telling friends, telling relatives, school absences), with alpha coefficients of 0.85 and 0.88, respectively.^20^ A higher total score indicates more T1DM-specific conflict within the family. Responses are “almost never,” “sometimes,” and “almost always.”

T1DM self-efficacy was measured using the 10-item Self-Efficacy for Diabetes Management scale (SEDM).^21^ The SEDM measures perceived self-efficacy for engagement in T1DM management tasks on a 10-point scale ranging from “not sure at all” to “completely sure.” Cronbach’s alpha for the scale is 0.90.^21^ A higher score indicates more T1D self-efficacy.

T1DM self-management was measured using the Self-Management of Type 1 Diabetes in Adolescents-Ireland Version (SMODA-I).^1^ The SMODA-I is a 52-item questionnaire that measures the following 5 subscales: collaboration with parents (range of scores 0-39), diabetes care activities (range of 0-45), diabetes problem-solving (range 0-21), diabetes communication (range 0-30), and goals (range 0-21). Higher scores are indicative of better performance in all subscales and alpha coefficients for each subscale range from 0.71 to 0.85.^1^ Responses are on a 4-point Likert type scale range from “never” to “always,” and for the goals subscale, responses range from “never a goal for me” to “always a goal for me.”

Division of family responsibility for T1DM management tasks was assessed using the 17-item Diabetes Family Responsibility Questionnaire (DFRQ).^22^ The DFRQ measures level of adolescent responsibility for T1D management tasks across 3 domains of regimen tasks, general health monitoring, and social presentation of diabetes. The alpha coefficient for the total scale is 0.84.^22^ Responses are: “I take responsibility for this almost all of the time,” “My parent(s) and I share responsibility for this about equally,” and “My parent(s) take responsibility for this almost all of the time.” Higher scores are indicative of more parental involvement in management tasks.

Activation was measured using the Patient Activation Measure-10-UK version (PAM-10-UK),^23^ comprising 10 items, responded to on a 5-point scale. The PAM-10-UK measures the perceived level of importance in the role of the respondent in the self-management of their own care. The PAM-10-UK was used in 1 previous study with children and adolescents (ages 8-17 years), and Cronbach’s alpha for this sample was 0.73.^17^

An overview of the measures is provided in Table 1.

**Table 1:** Overview of Instruments Included in Survey

Scale/subscale	Scoring	Items	α in current sample
Parent-Adolescent Communication Scale–Adolescent version^10^	5-point Likert scale ranging from “strongly disagree” to “strongly agree”		
Openness in communication	Higher scores indicate higher openness	10	.91
Problems in communication	Higher scores indicate less problems	10	.76
Total communication	Higher scores are indicative of better parent-adolescent communication	20	.89
Diabetes Family Conflict Scale–Revised^20^	3-point Likert scale with higher scores indicating more parent involvement in T1DM management; responses are “almost never,” “sometimes,” and “almost always”	19	.91
Self-Efficacy for Diabetes Management^21^	10-point Likert-type scale; higher scores indicate higher self-efficacy for T1DM management	10	.86
Self-Management of Type 1 Diabetes in Adolescents^1^	4- point Likert scale ranging from “never” to “always” and for the goals subscale ranging from “never a goal for me” to “always a goal for me”		
Collaboration with parents	Higher scores indicate more collaboration with parents	13	.87
Diabetes care activities	Higher scores indicate more performance of diabetes care activities	15	.69
Diabetes problem-solving	Higher scores indicate more and better problem-solving related to diabetes	7	.78
Diabetes communication	Higher scores indicate more communication about diabetes	10	.73
Goals	Higher scores indicate more diabetes goals	7	.75
Diabetes Family Responsibility Questionnaire^22^	3-point Likert scale; responses are “I take responsibility for this almost all of the time,” “My parent(s) and I share responsibility for this about equally,” and “My parent(s) take responsibility for this almost all of the time”; higher scores indicate more parent involvement in T1DM management	17	.81
Patient Activation Measure^23^	10-point Likert-type scale; four response options range from “disagree strongly to “agree strongly”; N/A response option also included	10	.76

Abbreviation: T1DM, type 1 diabetes mellitus.

### Statistical Analysis

Statistical analysis was carried out in IBM Statistics Package for Social Sciences version 25.0.^24^ Preliminary analyses were carried out to ensure no violation of the assumptions for hierarchical multiple regression analysis. Hierarchical multiple regression sought to determine what variables make the most unique contribution to self-management of T1DM, division of family responsibility for management tasks and activation, and was used to examine the association between 3 blocks of predictor variables: (1) demographic and clinical, (2) openness and problems subscale of the PACS and the DFCS-R, and (3) SEDM. Demographic and clinical variables (age, gender, duration of T1DM, and mode of insulin administration) identified by previous research as important influences of T1DM outcomes for adolescents were grouped together as context variables. The analysis sought to gain clarity on how self-management context (eg, age, gender, medical regimen, parent-adolescent communication, and family conflict) and self-management process (eg, self-efficacy) variables are related to self-management, activation, and division of family responsibility for T1DM management. Clinical and demographic variables were entered at step 1 (gender, age, T1DM duration, and mode of insulin administration). Parent-adolescent communication and family conflict variables were entered at step 2 (openness in parent-adolescent communication, extent of problems in parent-adolescent communication, and T1DM-specific family conflict). Finally, T1DM self-efficacy was entered at step 3.

The outcome variables for the hierarchical multiple regression analyses were each of the 5 subscales of the SMODA-I, the PAM-10-UK, and the DFRQ. Preliminary analyses were conducted to check for the presence of multicollinearity within the data and to ensure the assumptions of normality, linearity, homoscedasticity, and independence of residuals were met for multiple regression. The data set was also checked for influential cases. It is advised that influential cases are identified through evaluation of multiple methods.^25^ In the current analysis, standardized residuals, standardized DFBeta values, and leverage points measured using Mahalanobis distance were consulted. Cook’s distance was also assessed to evaluate the impact of an observation on overall model fit. Based on inspection of these values, 2 cases were identified as influential in the model predicting the DFRQ and the problem-solving subscale of the SMODA-I. These cases were excluded from these analyses.

## Results

A total of 166 survey packs were distributed to families; 113 adolescents ages 11 17 years (mean age 13.85 years, SD 1.78) and living with T1DM for 6 months and longer returned completed questionnaires. Most questionnaires were completed in hard-copy format (101 hard copy and 12 online). Where their last recorded A1C was known or reported (n = 101), most adolescents (n = 82) reported A1C higher than the blood glucose targets set by the International Society for Paediatric and Adolescent Diabetes for children, adolescents, and young adults (≤7%).^26^ Most adolescents administered insulin via an insulin pump (66%), with the remainder reliant on multiple daily injections (MDI). A breakdown of the demographic and clinical characteristics of participants is presented in Table 2.

**Table 2:** Demographic and Clinical Characteristics of Adolescents

Adolescent characteristics	
Gender, n (%)	
Male	58 (51%)
Female	55 (49%)
Age, n (%)	
11-13 y	55 (49%)
14-15 y	27 (24%)
16-17 y	31 (27%)
Mean	13.85 y
SD	1.78 y
Range	11-17 y
Age (y) at diagnosis	
Mean	8.19 y
SD	3.37 y
Range	1-16 y
BGM, n (%)	
Finger prick test only	30 (26%)
Flash glucose monitoring system and finger prick test	71 (63%)
Continuous glucose monitoring system and finger prick test	12 (11%)
Insulin administration, n (%)	
MDI	39 (34.5%)
Insulin pump	74 (65.5%)
Last A1C result, n (%)	
<6.5% (<48 mmol/mol)	7 (6.2%)
6.6% to 7.0% (49-53 mmol/mol)	12 (10.6%)
7.1% to 7.5% (54-58 mmol/mol)	20 (17.7%)
7.6% to 8.0% (60-64 mmol/mol)	23 (20.4%)
8.1% to 8.5% (65-69 mmol/mol)	25 (22.1%)
8.6% to 9.0% (70-75 mmol/mol)	6 (5.3%)
9.1% to 9.5% (76-80 mmol/mol)	3 (2.7%)
>9.5% (> 80 mmol/mol)	5 (4.4%)
Unknown	9 (8.0%)
Missing	3 (2.7%)
Family history of T1DM (parent with T1DM), n (%)	
Yes	6 (5.3%)
No	107 (94.7%)
Family history of T1DM (sibling with T1DM), n (%)	
Yes	15 (13.3%)
No	98 (86.7%)

Abbreviations: BGM, blood glucose monitoring; MDI, multiple daily injections; T1DM, type 1 diabetes mellitus.

Descriptive statistics, including means, standard deviations, and ranges for each measure reported on, are presented in Table 3. The values presented in Table 3 suggest that participants scored highly for openness in parent-adolescent communication (PACS), collaboration with parents (SMODA-I), performance of T1DM care activities (SMODA-I) and T1DM self-efficacy (SEDM) and had low scores for T1DM-specific family conflict (DFCS-R).

**Table 3.** Descriptive Information on Survey Responses

Scale/subscale	Mean	SD	Actual range (possible range)	N
PACS				
Openness	41.35	7.32	16-50 (10-50)	113
Problems in family communication	33.87	7.69	16-50 (10-50)	113
Total communication	75.21	13.36	34-97 (20-100)	113
DFCS-R	25.44	6.29	19-54 (19-57)	112
SEDM	72.21	16.28	36-100 (10-100)	112
SMODA-I				
Collaboration with parents	21.41	7.86	4-39 (0-39)	113
Diabetes care activities	32.98	5.26	20-43 (0-45)	113
Diabetes problem solving	15.07	4.54	2-21 (0-21)	113
Diabetes communication	18.00	4.94	6-28 (0-30)	113
Goals	15.83	3.13	7-21 (0-21)	113
DFRQ	31.22	4.91	21-43 (17-51)	112
PAM-10-UK	63.97	14.52	39-100 (0-100)	113
Frequency of PAM-10-UK activation levels				
Level :1 disengaged and overwhelmed	8 (7.1%)			
Level 2: becoming aware, but still struggling	31 (27.4%)			
Level 3: taking action	45 (39.8%)			
Level 4: maintaining behaviors and pushing further	24 (25.7%)			

Abbreviations: DFCS-R, Diabetes Family Conflict Scale-Revised–Youth Version; DFRQ, Diabetes Family Responsibility Questionnaire; PACS, Parent-Adolescent Communication Scale; PAM-10-UK, Patient Activation Measure-10-UK version; SEDM, Self-Efficacy for Diabetes Management scale; SMODA-I, Self-Management of Type 1 Diabetes in Adolescents-Ireland Version.

### Hierarchical Regression Analysis

The results of the hierarchical regression analyses are presented in Table 4.

**Table 4:** Hierarchical Multiple Regression Analyses Predicting Self-Management (SMODA-I), Family Responsibility (DFRQ), and Activation (PAM-10-UK)

	Collaboration with parents (SMODA-I)		Care activities (SMODA-I)		Problem-solving (SMODA-I)		Communication (SMODA-I)		Goals (SMODA-I)		Division of family responsibility (DFRQ)		Patient activation (PAM-10-UK)	
Predictor	*R*^2^ change	β	*R*^2^ change	β	*R*^2^ change	β	*R*^2^ change	β	*R*^2^ change	β	*R*^2^ change	β	*R*^2^ change	β
Step 1	.34**		.06		.34**		.01		.17**		.46**		.10*	
Gender		−.08		−.02		.07		−.02		.06		−.10		−.03
Age		−.51**		−.03		.32**		.05		.27**		−.66**		.29**
T1DM duration		.15		−.18		.03		.07		−.18		.13		−.07
Insulin mode		−.33**		.23*		.46**		−.04		.36**		−.23**		.16
Step 2	.07**		.13**		.01		.18**		.14**		.05*		.13**	
Gender		−.04		−.07		.06		−.09		−.002		−.06		−.04
Age		−.48**		.02		.32**		.10		.30**		−.65**		.34**
T1DM duration		.14		−.14		.03		.10		−.16		.12		−.07
Insulin mode		−.33**		.18		.46**		−.08		.35**		−.19*		.16
Openness in communication (PACS)		.01		.16		.09		.42**		.43**		−.03		.36**
Problems in communication (PACS)		.27**		.09		−.09		−.16		−.29**		.20*		.001
Family conflict (DFCS-R)		.09		−.25*		−.03		−.24*		−.16		.19*		−.01
Step 3	.003		.18**		.11**		.14**		.11**		.03*		.31**	
Gender		−.05		−.003		.12		−.03		.05		−.08		.05
Age		−.47**		−.04		.26**		.05		.25**		−.62**		.26
T1DM duration		.13		−.05		.12		.18		−.09		.08		.06
Insulin mode		−.31**		.03		.35**		−.21*		.24**		−.14		−.03
Openness in communication (PACS)		.03		.04		−.01		.31**		.33**		.02		.20*
Problems in communication (PACS)		.27**		.08		−.07		−.18		−.30**		.19		−.02
Family conflict (DFCS-R)		.08		−.15		.16		−.15		−.08		.10		.13
T1DM self-efficacy (SEDM)		−.06		.49**		.43**		.43**		.38**		−.20*		.65**
Total *R*^2^	.41		.37		.46		.33		.42		.53		.54	
N	111		111		109		111		111		108		111	

Abbreviations: DFCS-R, Diabetes Family Conflict Scale-Revised–Youth Version; DFRQ, Diabetes Family Responsibility Questionnaire; PACS, Parent-Adolescent Communication Scale; PAM-10-UK, Patient Activation Measure-10-UK version; SEDM, Self-Efficacy for Diabetes Management scale; SMODA-I, Self-Management of Type 1 Diabetes in Adolescents-Ireland Version; T1DM, type 1 diabetes mellitus.

* *P* < .05. **P < .01.

In the final model predicting collaboration with parents (SMODA-I subscale), younger age, use of MDI, and fewer problems in parent-adolescent communication were significant predictors. The final model explained 41% of the variance in collaboration with parents, *F*(8, 102) = 8.99, *P* *<* .001.

In the final model predicting TIDM care activities (SMODA-I subscale), T1DM self-efficacy was a significant predictor, with higher T1DM self-efficacy associated with more performance of T1DM care activities. The final model explained 37% of the variance in T1DM care activities, *F*(8, 102) = 7.48, *P* < .001.

In the model predicting problem-solving (SMODA-I), older age, use of an insulin pump, and higher T1DM self-efficacy were significant predictors of better problem-solving ability. Overall, the final model explained 46% of the variance in self-management problem-solving, *F*(8, 100) = 10.53, *P* < .001.

In the final model predicting T1DM communication (SMODA-I subscale), use of MDI, more openness in parent-adolescent communication, and higher T1DM self-efficacy were significant predictors. The final model explained 33% of the variance in T1DM communication, *F*(8, 102) = 6.29, *P* < .001.

In the model predicting self-management goals (SMODA-I subscale), age, use of an insulin pump, more openness in parent-adolescent communication, more problems in parent-adolescent communication, and higher T1DM self-efficacy were significant predictors of more T1DM self-management goals. The final model explained 42% of the variance in self-management goals, *F*(8, 102) = 9.37, *P* < .001.

In the model predicting division of responsibility for T1DM management (DFRQ), younger age and lower T1DM self-efficacy were associated with more parent involvement in management. The final model explained 53% of the variance in family responsibility for management, *F*(8, 99) = 13.87, *P* < .001.

In the model predicting activation (PAM-10-UK), more openness in parent-adolescent communication and higher T1DM self-efficacy significantly associated with more activation. The final model explained 54% of the variance in activation, *F*(8, 102) = 15.04, *P* < .001.

## Discussion

To our knowledge, this is the first study to investigate the interrelationships between family context variables (parent-adolescent communication, T1DM-specific family conflict), self-management process (T1DM self-efficacy), and self-management behaviors/proximal outcomes (self-management of T1DM, division of responsibility for T1DM management, activation). This study revealed that openness in parent-adolescent communication, extent of problems in parent-adolescent communication, and family conflict differentially predict activation, how responsibilities for management tasks are divided between parents and adolescents, and aspects of self-management. Findings also highlighted that self-efficacy contributes to explaining activation, division of responsibility for management tasks within the family, and self-management, except for collaboration with parents.

The findings that family context, more specifically, parent-adolescent communication and family conflict, is related to self-management provide unique insights into these specific aspects of family functioning and their relationship to self-management. Whereas previous research found that family behaviors and family functioning are related to T1DM management during adolescence,^27-30^ this study considers, for the first time, parent-adolescent communication specifically and its relationship to self-management more broadly as a dynamic and proactive process rather than engagement with a specific management behavior or blood glucose targets. The specific focus on parent-adolescent communication in this study offers a new perspective in that much of the research in this field to date has predominantly considered the relationships of family conflict and general family functioning to T1DM management.^6,8,13^ That different aspects of parent-adolescent communication were related with distinct aspects of self-management in this study reinforces the need for continued research to consider the precise nature of parent-adolescent interactions that are relevant to T1DM outcomes in adolescence.

The association identified between more problems in parent-adolescent communication and greater adolescent self-management goals was unexpected given that previous studies highlight the links between positive parental interactions with more optimal self-management and engagement with T1DM management behaviours.^31,32^ Furthermore, more problems in parent-adolescent communication were also associated with less collaboration with parents. It may be that the presence of problems in communication may act as a driving factor, encouraging increased adolescent autonomy, or may be a feature of adolescents striving for more independence in their management. Adolescence is characterized as a period for increasingly separating from parents’ influence, which can introduce conflict into parent-adolescent interactions and relationships.^33^ As adolescents are gaining autonomy in all aspects of life, this can present specific conflict and problems in communication with parents.^34^ Previous research suggests that more family conflict may be reported as adolescents become more self-reliant in their T1DM management.^35^ It is possible that this period of striving toward the goals of independent management is characterized by more problems in communication with parents. However, given that the data are cross-sectional, this limits the conclusions that can be drawn, and additional longitudinal investigation into the role and nature of problems in parent-adolescent communication and gaining independence in self-management is necessary.

The findings of this study also highlighted that adolescent-perceived openness in parent-adolescent communication is important for T1DM goals, communication about T1DM, and greater activation. This study on openness in parent-adolescent communication advances existing research that found that more disclosure and honesty in parent-adolescent interactions has been associated with better T1DM management.^36,37^ When adolescents perceive parental behaviors as warm and caring, this contributes to more adolescent participation in self-care behaviors.^38^ Honesty, warmth, and interactions with high levels of disclosure could be considered to share some of the characteristics of openness in communication. However, this is the first study to specifically measure openness in parent-adolescent communication in adolescents living with T1DM. Findings suggest the presence of openness in parent-adolescent interactions might contribute to better self-management outcomes in adolescents. Given that dimensions of parent-adolescent communication impact differentially on aspects of self-management suggests openness and problems in parent-adolescent communication as they relate to specific aspects of self-management of T1DM in adolescence warrant further investigation.

However, the finding that family conflict was no longer associated with performance of T1DM care activities and division of responsibility for management tasks contrasts with previous research that found that more family conflict and less self-efficacy was related to lower engagement with self-management behaviors and glycemic variability.^39^ Considering that parent-adolescent communication remained significantly associated but T1DM-specific family conflict did not in the final models might suggest these constructs were more important among the adolescent respondents in this study. However, it is worth noting that the mean score for conflict among current respondents was indicative of low conflict.

The finding that parent-adolescent communication and self-efficacy are associated with T1DM self-management is supported by theories of self-efficacy. Receiving feedback on performance can enhance self-efficacy and perceptions of competence in completing a specific task.^40^ The association between self-efficacy and positive engagement with T1DM self-management behaviors is well established.^21-39^ The findings of this study that more openness in communication and higher self-efficacy contribute to more self-management communication, self-management goals, and activation add to knowledge on what constitutes helpful parent-adolescent interactions that promote adolescent engagement with self-management. When parents are perceived as supportive toward the adolescent’s T1DM management, this can reinforce engagement with positive management behaviors and increase perceptions of self-efficacy for carrying out T1DM management.^12^ Furthermore, when adolescents are clear on their parents’ expectations and feel respected by parents, this contributes to motivation to engage with self-management behaviors of their own decision.^5^ This study advances this evidence to suggest that parent-adolescent communication is a specific domain of family function that could be targeted along with self-efficacy to improve engagement with and motivation toward self-management.

This study also investigated, for the first time, associations between T1DM self-efficacy, parent-adolescent communication, and T1DM-specific conflict and levels of patient activation. Activation is important given its focus on how engaged individuals are in the management of their care. Furthermore, activation has been associated with improved health outcomes in adults.^41^ This study, for the first time, measures activation in adolescents living with T1DM. It is also the first study to examine the relationship of any aspect of family functioning to activation. Openness in parent-adolescent communication was associated with activation even when T1DM self-efficacy was also accounted for. This suggests that promoting open communication between parents and adolescents may lead to increases in activation. Changes in activation have been linked with performance of self-management behaviors in adults with chronic illness.^23^ The findings of this study are also comparable with the strong correlations previously observed for self-efficacy and activation in a small sample of children and adolescents living with a chronic illness or a complex health care need that required hospitalization.^17^ However, the measure of activation (PAM-10-UK) has not been widely used in pediatric settings, and thus, there is a need for continued research that evaluates activation in adolescent chronic illness, including T1DM.^17,42^ The findings of this study indicate that activation is a construct that should be considered in conjunction with self-management in adolescents living with T1DM.

The findings of this study are important because they indicate there may be benefits to encouraging adolescents and parents to employ communication strategies that are open to ensure that both adolescents and parents have clear expectations with respect to their involvement and roles in managing T1DM. Employing open communication strategies should also reduce secrecy surrounding instances where adolescents are not engaging with self-management and contribute to the formation of an environment where the adolescent openly shares information about their T1DM management with their parents. Family-focused interventions should seek to encourage parents to utilize communication strategies about T1DM that are adolescent-centered and supportive of adolescent T1DM management self-efficacy because these may lead to more optimal negotiation of responsibilities for T1DM self-management in adolescence.

There are some limitations to this study to consider. First, given the cross-sectional design, the relationships identified do not imply causality. Future studies may wish to consider a longitudinal design to follow up with adolescents during the period of transitioning responsibilities for self-management of T1DM. Longitudinal research may acquire an increased understanding of the relationships between parent-adolescent communication and self-management over time. Further research is also needed to investigate whether there is an association between parent-adolescent communication, T1DM-specific family conflict, and T1DM self-efficacy and glycemic stability over time. Second, this study measures only 2 dimensions of parent-adolescent communication. Future studies might seek to quantitatively assess other dimensions of family function and communication (eg, affective involvement) in addition to openness, extent of problems in parent-adolescent communication, family conflict related to T1DM, and their impact on self-management. Third, self-selection bias cannot be ruled out because the sample is limited to adolescents who voluntarily completed the survey. Fourth, this study sample reported low levels of family conflict, and consequently, results may not be reflective of adolescents experiencing high levels of family conflict.

## Conclusions

This study highlights characteristics of parent-adolescent communication, specifically, openness and extent of problems, as perceived by adolescents, which are associated with self-management. Understanding how communication potentially features in the negotiation and navigation of management responsibilities during adolescence is critical, especially given the difficulties with T1DM management that occur over the course of adolescence. The findings are important because they can inform future family-focused self-management interventions to improve T1DM outcomes for adolescents living with T1DM.
